# *Wolbachia* protects *Drosophila melanogaster* against two naturally occurring and virulent viral pathogens

**DOI:** 10.1038/s41598-023-35726-z

**Published:** 2023-05-25

**Authors:** Gaspar Bruner-Montero, Francis M. Jiggins

**Affiliations:** 1 Department of Genetics, University of Cambridge, Cambridge, CB2 3EH UK; 2 Coiba Scientific Station, City of Knowledge, 0843-03081 Clayton, Panama

**Keywords:** Evolution, Entomology

## Abstract

*Wolbachia* is a common endosymbiont that can protect insects against viral pathogens. However, whether the antiviral effects of *Wolbachia* have a significant effect on fitness remains unclear. We have investigated the interaction between *Drosophila melanogaster, Wolbachia* and two viruses that we recently isolated from wild flies, La Jolla virus (LJV; *Iflaviridae*) and Newfield virus (NFV; *Permutotetraviridae*). Flies infected with these viruses have increased mortality rates, and NFV partially sterilizes females. These effects on fitness were reduced in *Wolbachia-*infected flies, and this was associated with reduced viral titres. However, *Wolbachia* alone also reduces survival, and under our experimental conditions these costs of the symbiont can outweigh the benefits of antiviral protection. In contrast, protection against the sterilizing effect of NFV leads to a net benefit of *Wolbachia* infection after exposure to the virus. These results support the hypothesis that *Wolbachia* is an important defense against the natural pathogens of *D. melanogaster.* Furthermore, by reducing the cost of *Wolbachia* infection, the antiviral effects of *Wolbachia* may aid its invasion into populations and help explain why it is so common in nature.

## Introduction

Microbial endosymbionts can be beneficial partners that provide a source of evolutionary innovation to their hosts, or parasites that spread through host populations despite reducing the fitness of the individuals they infect. This is illustrated by *Wolbachia*, a maternally transmitted endosymbiotic bacterium that infects about half of insect species^1–3^. Classically, *Wolbachia* was considered a reproductive parasite that manipulated the reproduction of its hosts to promote its transmission to future generations. Some strains distort host sex ratios towards females—the sex that transmits the symbiont to its offspring—by inducing parthenogenesis, feminizing genetic males or killing males^4^. Others induce a sperm-egg incompatibility called cytoplasmic incompatibility (CI), favoring *Wolbachia-*infected females in the population^4^. However, some strains of *Wolbachia* are thought to cause a net increase in host fitness and can therefore be considered as mutualists. In the bedbug *Cimex lectularius*, *Wolbachia* is an obligate nutritional mutualist, synthesizing B vitamins missing from the host’s diet^5^. Similarly, many filarial nematodes have an obligate mutualism with *Wolbachia*^6^.

How frequently *Wolbachia* symbioses benefit arthropod hosts is unclear^7^. Obligate symbioses, such as B vitamin synthesis in bedbugs, are likely rare. However, many more *Wolbachia* infections may be facultative mutualists, where the symbiont can be beneficial but not essential for host survival or reproduction. A compelling argument that such benefits are widespread comes from the observation that a large proportion of *Wolbachia* infections produce no detectable reproductive manipulation. While in some cases such symbionts may have recently lost a reproductive manipulation and be in the process of being lost from the population, in others their frequency is increasing and they must therefore confer a fitness benefit^8,9^. This is exemplified by the *Wolbachia* strain *w*Au in *Drosophila simulans,* which can invade host populations without manipulating host reproduction^10^. Consistent with this, a number of studies have reported that *Wolbachia-*infected insects have increased survival or fecundity^7^. For example, on certain diets *Wolbachia* buffers *Drosophila melanogaster* against nutritional stress^11^. Some of these may be ‘Jekyll and Hyde’ symbionts that simultaneously act as reproductive parasites and beneficial mutualists^12^. This can be important as strains that induce CI can only invade populations when their frequency in the population becomes sufficiently high to offset imperfect maternal transmission and infection costs^13^. Recent data suggested that CI-inducing *Wolbachia* can sometimes spread from very low frequencies, suggesting these strains also carry a fitness benefit^10^.

One mechanism by which *Wolbachia* may provide a fitness advantage is by protecting hosts from pathogens. Multiple strains of *Wolbachia* have been shown to defend insects against viruses^14–16^. When these were artificially transferred to *Aedes aegypti*, the primary vector of human viral pathogens, they reduce the transmission and replication of Dengue virus^17^. Although the antiviral mechanisms derived from *Wolbachia* are poorly understood, it has been argued that *Wolbachia* competes for the host's cellular resources, limiting and blocking the resources required by viral pathogens^18–20^. Whatever the mechanism of antiviral protection, the level of antiviral protection of *Wolbachia* depends on its density and varies between strains^14,21,22^.

Although *Wolbachia* decreases viral load in *Drosophila* under laboratory conditions, the importance of this under field conditions is less clear, leading some to argue that the antiviral effects of *Wolbachia* may have little effect on fitness in natural populations^7^. We recently found that in wild *Drosophila* populations *Wolbachia-*infected flies were less likely to be infected by viruses^23^. However, most naturally occurring *Drosophila* viruses have not been isolated^24^, which prevents laboratory studies from experimentally confirming antiviral effects. Furthermore, without viral isolates it is not possible to test whether the benefits of antiviral protection outweigh the cost of *Wolbachia* infection itself. Therefore, to expand our understanding of the interaction between *Drosophila*, *Wolbachia* and virus, we investigated the effect of *Wolbachia* on La Jolla virus (LJV) and Newfield virus (NFV), which we recently isolated from natural populations of *D. melanogaster*^25^. These are both positive-sense single-stranded RNA viruses. NFV has a small genome size (4.7 kb) and belongs to an unclassified genus within the *Permutotetraviridae* family. LJV has a larger genome (9.7 kb) and belongs to an unclassified genus within the *Iflaviridae* family. LJV is one of the most abundant viruses in populations of *D*. *simulans* and *D. melanogaster*, while NFV is less common^24^. Here we report that *Wolbachia* provides some protection against these viruses, reducing both viral titre and virulence.

## Results

### *Wolbachia* reduces viral titre

**Figure 1:**
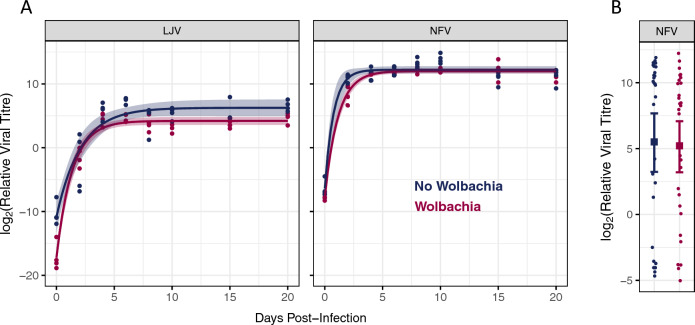
The effect of *Wolbachia* on viral titre. (**A**) Viral titre in flies with and without *Wolbachia* that were inoculated with LJV or NFV. The line is a three-parameter asymptotic exponential growth curve, the shaded areas are 95% confidence intervals, and each point is an RNA extraction from 10 flies. (**B**) Viral titre 10 days post oral infection with NFV. Each point represents the virus titre of a single fly. The square is the mean and error bars 95% bootstrap confidence interval. Viral titres were estimated by quantitative PCR relative to the concentration of *RpL32* mRNA. Virus was detected in all samples. Male flies were added 3–5 days post eclosion.

To investigate the effect of *Wolbachia* on viral titre, 3–5 day old virgin female flies were inoculated with LJV or NFV and titres estimated at eight timepoints from 0 to 20 days. Both viruses initially replicated rapidly and by ~ 4 days post-infection (dpi) the titre plateaued (Fig. 1A). NFV titres increased faster than LJV and reached a higher level. *Wolbachia* significantly reduced the titre of both viruses (ANOVA, LJV: Time × *Wolbachia*, *F* = 3.37, d.f. = 7,48, *p* = 0.005; NFV: Time × *Wolbachia*, *F* = 3.71, d.f. = 7,48, *p* = 0.003). LJV titres were mostly reduced at later timepoints when the titre had stabilised, while NFV titres were reduced earlier in the infection (Fig. 1A). Averaging across timepoints, the titre of LJV was 4.2 times greater in *Wolbachia-*free flies while NFV titres were 1.6 times greater.

To examine the effects of *Wolbachia* on the virus after oral infection we fed adult flies on yeast paste that was contaminated with the virus. Ten dpi, NFV could be detected in all the flies that had been fed the virus, but there was no significant effect of *Wolbachia* on the viral titre (Fig. 1B; Welch two sample* t* test: *t* = 0.19, d.f. = 54.8, *p* = 0.85). However, there was over 100,000-fold variation in the titres of individual flies, so we have limited statistical power (Fig. 1B). Flies were also fed LJV, but only 5 of 59 flies had detectable levels of virus 10 dpi (the remainder of the flies had no PCR amplification after 40 cycles).

### *Wolbachia* increases lifespan after infection with LJV but not NFV

**Figure 2:**
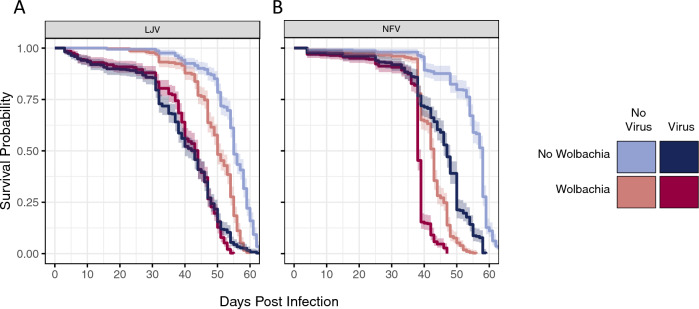
The effect of virus and *Wolbachia* infection on mortality. Shaded area is the 95% confidence interval. Each line is estimated from 307 to 383 flies. Male flies were infected 3–5 days post eclosion.

*Wolbachia* has an effect on viral titre (‘interference’), but it is the effects on fitness (‘protection’) that will affect the evolution and population dynamics of the symbiont. We therefore investigated survival and fecundity. On their own, both *Wolbachia* and viral infection substantially reduce the survival of flies. In the absence of *Wolbachia*, NFV shortened the lifespan of flies by a mean of 10 days (Fig. 2B; Cox Mixed-Effect model, Tukey’s Test: z = 16.51, *p* < 0.001), while LJV reduced mean lifespan by 15 days (Fig. 2A; Cox Mixed-Effect model, Tukey’s Test: z = 21.77, *p* < 0.001). As has been reported previously^21^, in both experiments we found that *Wolbachia* infection in virus-free flies reduces lifespan (Cox Mixed-Effect model, Tukey’s Tests; Fig. 2A: z = 23.31, *p* < 0.001; Fig. 2B: z = 22.79, *p* < 0.001). However, the magnitude of the effect differed in the two experiments (Fig. 2A vs 2B). In both cases the *Wolbachia*-free flies lived a mean of 54 days. However, the *Wolbachia*-infected flies lived a mean of 42 days in the first experiment and 49 days in the second experiment. The cause of this is unknown as the fly lines and experimental protocol were not changed.

To test whether *Wolbachia* reduces the rate at which the virus kills flies, we compared the rate of virus-induced mortality in *Wolbachia*-infected and *Wolbachia*-free flies. We found that *Wolbachia* significantly reduced the rate at which both viruses kill flies. In the case of NFV, the virus reduced mean lifespan by 5 days in *Wolbachia*-infected flies as opposed to 10 days in *Wolbachia*-free flies (Fig. 2B; Cox Mixed-Effect model, *Wolbachia* × virus: *z* = 3.69, *p* < 0.001). In the case of LJV, the virus reduced mean lifespan by 9 days in *Wolbachia*-infected flies as opposed to 15 days in *Wolbachia*-free flies (Fig. 2A; Cox Mixed-Effect model, *Wolbachia* × virus: *z* = 7.17, *p* < 0.001).

Finally, we examined whether this reduction in the rate at which the virus kills flies outweighs the cost of carrying *Wolbachia*—do virus-infected flies benefit from the presence of *Wolbachia*? In the case of NFV this is not the case, as flies infected with both *Wolbachia* and virus died on average 8 days before those infected with NFV but not *Wolbachia* (Fig. 2B; Cox Mixed-Effect model, Tukey’s Test: *z* = 20.44, *p* < 0.001). In contrast, there was a small and marginally significant net benefit of *Wolbachia* in flies infected with LJV, with *Wolbachia* extending the life of virus infected flies by a mean of 0.8 days (Fig. 2A; Cox Mixed-Effect model, Tukey’s Test: *z* = 2.78, *p* = 0.02). Note that the cost of *Wolbachia* itself was lower in the LJV experiment (see above), so this may in part be causing this apparent difference between the viruses. In summary, our results show that while *Wolbachia*-infected flies may suffer fewer virus-induced deaths, these benefits can be outweighed by the cost of *Wolbachia* infection itself.

### *Wolbachia provides partial* protection against sterilisation by* NFV after oral infection*

**Figure 3:**
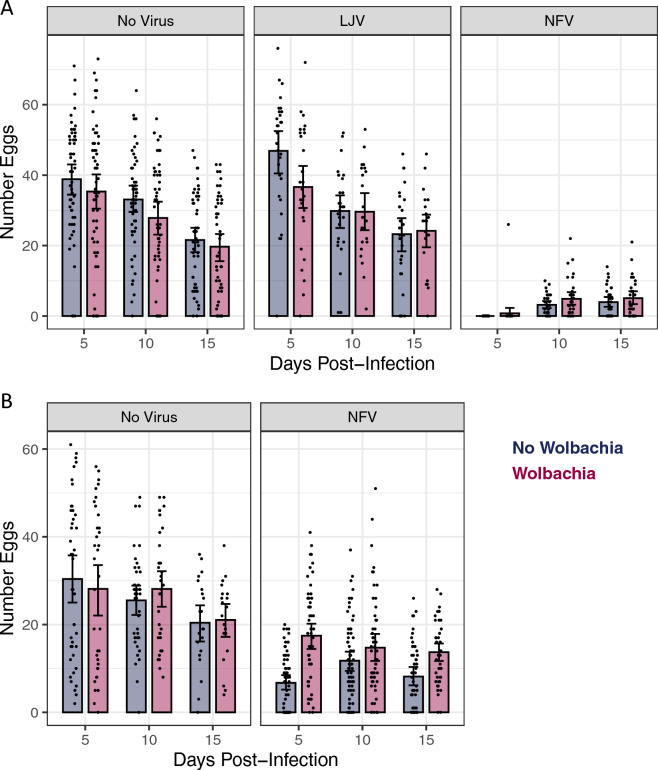
The effect of virus and *Wolbachia* infection on female fecundity. Egg production in flies with and without *Wolbachia* that had been (**A**) inoculated with LJV or NFV or (**B**) fed NFV. Each dot represents the number of eggs laid per single female over 24 h. The bars are means, and error bars 95% confidence intervals. Flies were infected 3–5 days post eclosion.

When female flies were inoculated by NFV they suffered a large reduction in their fecundity, which is in-line with our previously reported results^25^ (Fig. 3A; GLMM, Tukey’s post-hoc contrasts uninfected versus NFV: *p* < 0.0001 at all timepoints). At 5 dpi NFV transiently sterilised flies, with only one fly laying any eggs (Fig. 3A). However, they gradually recovered, with the NFV-infected flies having an 85% reduction in fecundity at 10 dpi, and a 78% reduction at 15 dpi (Fig. 3A; estimates from back-transformed GLMM coefficients). In contrast, LJV did not significantly affect female fecundity at any timepoint (Fig. 3A; GLMM, Tukey’s post-hoc contrasts uninfected versus LJV: *p* > 0.09 at all timepoints). If only the virus-free flies are analysed, there is no significant effect of *Wolbachia* on fecundity (Fig. 3A, main effect *Wolbachia*: χ^2^ = 3.3, d.f. = 1, *p* = 0.07).

When flies were infected orally with NFV, the virus also strongly reduced the number of eggs that females laid (Fig. 3B). Unlike flies that had been inoculated with NFV, oral infection did not completely sterilise flies at early timepoints (Fig. 3B). Nonetheless, averaged across the three timepoints we studied the virus caused a 64% reduction in the number of eggs laid by *Wolbachia*-free flies (Fig. 3B; estimate from back-transformed GLMM coefficients). *Wolbachia* significantly reduced this impact of NFV on female fecundity (Fig. 3B; *Wolbachia* × virus × dpi: χ^2^ = 15.1, d.f. = 2, *p* = 0.0005)—in *Wolbachia*-infected flies, NFV reduced the number of eggs laid by 34% (estimate from back-transformed GLMM coefficients and averaged across timepoints).

## Discussion

As *Wolbachia* infects about half of insect species^3^ and many strains protect their hosts against viruses^14,26^, the symbiont may be a major antiviral defence in nature. However, insects harbour highly diverse communities of viruses and only a handful have had their interaction with *Wolbachia* described. With this in mind, we investigated two viruses what we recently isolated from wild *D. melanogaster.* One of these—LJV—is one of the most common viruses in the field, infecting slightly under 10% of wild flies^24^. We found that these viruses had lower titres and reduced virulence when flies were *Wolbachia-*infected. This supports the notion that *Wolbachia* has broad-spectrum antiviral effects against distantly related RNA viruses^14^.

Might this mean that *Wolbachia* is sometimes a mutualist, spreading through populations by protecting insects against their viral pathogens? These viruses both substantially reduce lifespan, although the effects are largely in later life when flies will have passed their reproductive peak. Furthermore, NFV near sterilises infected females. Therefore, there will be a selective advantage to any factor providing resistance. However, *Wolbachia* infection itself also carries a substantial cost in *D. melanogaster*^21^, so to be a mutualist the benefits of antiviral protection need to outweigh these costs. For LJV-infected flies, the effect of *Wolbachia* largely negated any benefits from antiviral effects—the significant net increase in lifespan was less than a day and so should be treated with caution. For NFV, the main effect on fitness is likely the partial sterilisation of female flies, and here again *Wolbachia* carries a net benefit in our experiment. While these results suggest *Wolbachia’*s antiviral effects may have significant effects on fitness in nature, they cannot be directly extrapolated to the wild. First, while the large majority of *D. melanogaster* in the wild are likely infected by viruses^23,27,28^, the most abundant viruses are thought to be less virulent than LJV and NFV^27^, so there may be correspondingly smaller fitness benefits to any protection that *Wolbachia* provides against these viruses. Second, our laboratory measurements of fitness components may differ greatly from the wild. For example, infection route, dose and resource availability are all likely to alter the fitness effects of both *Wolbachia* and virus infection. Even within two experiments we report there was a marked difference in the effect of *Wolbachia* on survival, suggesting that subtle changes in conditions may shift the balance of costs and benefits. Quantitative estimation of the fitness of *Wolbachia-* and virus-infected flies will need to be done in the field.

While it is unknown if the antiviral effects of *Wolbachia* are sufficient to make it a mutualist that increases the fitness of flies, they may go some way to offsetting the fitness cost of the symbiont. Aside from studies like this showing that *Wolbachia* reduces the harm of virus infection under laboratory conditions, we recently found that in the wild *Wolbachia-*infected flies were less likely to be infected by viruses^23^. Even if there remains a net cost to *Wolbachia* infection, by reducing the cost of symbiont infection, the threshold prevalence that *Wolbachia* must reach to invade a population will be reduced^10^, making invasion more likely. Furthermore, the fitness benefits of being protected against viruses likely vary greatly through space and time. Therefore, a viral epidemic may provide a temporary benefit to *Wolbachia* infected flies, driving the symbiont prevalence above the invasion threshold. Once the viral prevalence declines again, *Wolbachia* may be maintained by cytoplasmic incompatibility. In conclusion, we have shown that *Wolbachia* can protect flies against infection with two naturally occurring *Drosophila* viruses under laboratory conditions. If these results hold in natural infections, this may be an important factor in shaping the dynamics of the symbiont within populations.

*Wolbachia* is known to have very different effects on the titres of different viruses^14,29^. For example, it causes far greater reductions in the titres of DCV than FHV in *D. melanogaster* and yet provides large increases in survival after infection with both viruses^14,29^*.* The reductions in viral titre that we observed were comparatively modest, and considerably smaller than those seen for DCV^29^. As was the case for FHV^29^, there was nonetheless substantial reductions in viral virulence. Whether these differences in titre are sufficient to cause the differences in survival and fecundity, or whether this is an effect on tolerance (a reduction in harm for a given pathogen load) is unknown.

## Materials and methods

### Fly husbandry and virus isolates

The *D*. *melanogaster* lines were kindly provided by Luis Teixeira. *Wolbachia*-infected flies with the strain wMelCS_b and its *Wolbachia*-free isogenic background DrosDel w^1118^ (w^1118^ iso)^21,30^ were used in the experiments. These lines were created when the *Wolbachia* variant wMelCS_b was introduced into the cytoplasm of DrosDel w^1118^ isogenic line background by chromosome substitution using balancers for the 1st, 2nd, and 3rd chromosomes—the 4th chromosome was not substituted^21^. Flies were maintained in glass vials (~ 28.5 × 95 mm) with standard cornmeal food (1200 ml water, 13 g agar, 105 g dextrose, 105 g maize, 23 g yeast, 35 ml Nipagin 10% w/v), and incubated at 25 °C and ~ 70% humidity.

Two RNA viruses that had been isolated from wild populations of *D*. *melanogaster* and used in this study. LJV isolate GBM-15052019-4-305 was isolated from Gialousa, Cyprus, and NFV isolate GBM-09102019-1-393 was isolated from Cambridge, UK^25^.

### Virus production

All virus isolates were cultured in DL2-B2 cells. DL2-B2 cells are Schneider *Drosophila* Line cells (DL2) stably transfected with the Flock House virus B2 protein, which is a strong suppressor of RNAi and facilitates the infection of the virus^25^. DL2-B2 cells were grown in plastic culture flasks containing Schneider medium supplemented with 10% v/v heat-inactivated fetal bovine serum (FBS) and streptomycin 100 µg/ml and penicillin 100 U/ml to inhibit bacteria and fungus contamination. To preserve aliquot stocks for the experiments, 2 ml of cell culture was added to a conical tube (Falcon™) and briefly spun (1000 g for 2 min) to remove cell’s debris, then the supernatant was aliquoted (10 µl) into sterilized 0.2 ml PCR tubes and stored at − 80 °C. The viral concentration of the isolates was estimated using the TCID50 ml^−1^ method^31^. The aliquot stocks were thawed immediately before each pricking assay on ice and diluted in sterilised Ringer’s solution to standardise the concentration of the virus isolates to 1 × 10^5^ TCID50 ml^−1^.

### Viral infection protocol

Flies were anesthetized on a CO_2_ pad and then pricked on the dorsolateral thorax under a stereomicroscope using a needle (Austerlitz Insect Pin) dipped into a Ringer’s solution containing 1 × 10^5^ TCID50 ml^−1^ viral titre. To avoid cross-contamination between viruses, different CO_2_ pads and needles were used for each virus. These utensils were kept in independent plastic bags and cleaned with Virkon^®^ (5% w/v) and ethanol (70% v/v) frequently. After infection, infected flies were kept in independent trays for each virus treatment to avoid cross-contamination. Unless otherwise mentioned, flies were transferred every 3 days to new vials with fresh food cornmeal and incubated at 25 °C over the course of the experiments.

### Oral infection protocol

To infect orally the flies, a cohort of 3–5 day old virgin adult females was transferred into an empty vial without food containing a damp towel paper for 24 h at 25 °C. The next day, the starved females were transferred into a vial containing 300 µl of yeast paste (25% w/v yeast powder, 5% v/v vegetable red dye, and 10^5^ TCID50 of one of the viruses and incubated at 25 °C. Another cohort of females was transferred into vials with Ringer’s solution (25% w/v yeast powder and 5% red dye v/v) as a control. To confirm that the flies ingested the virus solution, the next day flies' gut was checked under a stereoscopic microscope. Flies with red-stained intestines were selected for the experiment and transferred into new vials with fresh cornmeal food.

### Virus quantification

The total RNA of the homogenized flies was extracted using the chloroform isopropanol method following the manufacturer’s protocol (Life Technologies). One µl of RNA per sample was reverse-transcribed with Promega GoScript reverse transcriptase using random hexamer primers, and then diluted 1:10 with nuclease-free water. qRT–PCR was performed on an Applied Biosystems StepOnePlus system using Sensifast Hi-Rox Sybr kit (Bioline) with 1 µl of complementary DNA (cDNA) per sample was used to quantify the viral load using specific primers for LJV LaJolla1_foward (5’-CGGACCAGAGTGTAGCCAAG-3), and LaJolla1_reverse (5’-AGTGCCATCCAYCGATTTGT-3’), and NFV NewfieldVirus_2_forward (5’-TTGATGATGTCGCCACGAGA-3’), NewfiledVirus_2_reverse (5’-CATTCGCCGAGACCTCCATC-3’). The fly gene *RpL32* was used to normalize the expression using primers RpL32_forward (5’-TGCTAAGCTGTCGCACAAATGG-3’) and RpL32_reverse (5’-TGCGCTTGTTCGATCCGTAAC-3’)^32^. The qRT-qPCR was performed with the following PCR cycle: 95 °C for 2 min followed by 40 cycles of 95 °C for 5 secs followed by 60 °C for 30 secs. Two technical replicates per qRT-PCR reaction were carried out per sample with both the viral and reference genes. To estimate the relative viral load, the formula ΔCt = Ct_*RPL32*_–Ct_target_virus_ was used, where Ct is the mean Ct value of the technical replicates performed on each target sequence.

### *Wolbachia* effect on viral titre

To evaluate the effect of *Wolbachia* on viral titre, flies with and without *Wolbachia* were infected with LJV and NFV. Two male and 2 female flies with or without *Wolbachia* were transferred into 80 vials (n = 160 vials total), representing 80 biological replicates, containing standard diet and incubated at 25 °C. On 2 days into fresh vials and discarded on day 4. After 2 weeks, 10 adult males (3–5 days old) with *Wolbachia* (n = 80) or without *Wolbachia* (n = 80) were transferred into a vial with fresh cornmeal food. Then, 40 vials per *Wolbachia* treatment were pricked with LJV or NFV. Eight vials were pricked with Ringer’s solution (control). Zero (immediately post-infection), 2, 4, 6, 8, 10, 15, 20 days post infection (dpi) four vials of *Wolbachia-*infected and *Wolbachia-*free flies were anaesthetized using CO_2_ and transferred into four Eppendorf tube (2 ml) containing beads. Four control vials were collected on day 0 and 20 dpi. Every tube was chilled on ice for 10–15 min and 250 µl of TRIzol^®^ Reagent (Invitrogen) was added. Immediately, tubes were homogenised using a Qiagen TissueLyser II and stored at − 80 °C. For each tube, the total RNA was extracted, and the relative virus concentration was estimated as mentioned above.

### Lifespan experiment

To evaluate the effect of *Wolbachia* on the lifespan of flies infected with a virus, flies with and without *Wolbachia* were infected with LJV and NFV. For each virus, forty-four vials with flies (2 males and 2 females) per *Wolbachia* treatment (*n* = 88 vials total) were set up and the parental flies discarded as in the experiment above. After 2 weeks, 20 adult males (3–5 days old) with *Wolbachia* (*n* = 44) and without *Wolbachia* (*n* = 44) were transferred into a vial with fresh cornmeal food. Twenty-two of these vials per *Wolbachia* treatment were pricked with one of the viruses or Ringer’s solution (control). The experiment was performed for each virus independently. The number of dead flies per vial was monitored daily until the last fly was dead. Mortality on day 1 was attributed to the damage induced by the needle, and the flies that died on day 1 were ignored in our survival analyses.

### Fecundity assay

To investigate the effect of *Wolbachia* on fecundity after viral infection, female flies with and without *Wolbachia* were infected with LJV and NFV. Sixty vials with flies (2 males and 2 females) per *Wolbachia* treatment (*n* = 120 vials total) were set up and the parents were discarded as mentioned above. One virgin female and 2 males (3–5 days old) with *Wolbachia* (*n* = 60) and without *Wolbachia* (*n* = 60) were transferred into a vial with cornmeal food with live yeast sprinkles. Females from 20 vials per *Wolbachia* treatment were pricked with NFV, LJV, or Ringer’s solution (control). Males were not infected to avoid any influence on mating success. To quantify the number of eggs produced, flies were transferred into new vials without live yeast sprinkles with fresh food allowed to lay eggs for 24 h. The number of eggs was quantified under a stereomicroscope at 3-time points, between 4–5, 9–10, and 14–15 dpi. The experiment was repeated under identical conditions.

### Fecundity assay after oral infection

To investigate the effect of *Wolbachia* on fecundity after oral viral infection, female flies with and without *Wolbachia* were infected with NFV. This experiment was performed as the abovementioned fecundity assay with the exception that females were infected orally. Virgin females (3–5 days old) with *Wolbachia* and without *Wolbachia* were orally infected with NFV or Ringer’s solution (control) and transferred into new vials with fresh food and 2 males 3–5 days old. The number of eggs produced was quantified at 3-time points as the experiment above.

### *Wolbachia* effect on viral titre after oral infection

To investigate the antiviral effects of *Wolbachia* after oral viral infection, female flies with and without *Wolbachia* were infected with LJV and NFV. Adult females (n = 30 each) 3–5 days old were orally infected with NFV, LJV, or Ringer’s solution per *Wolbachia* treatment, and transferred into new vials with fresh food. Ten days after oral infection, the total RNA of single female flies was extracted, and the relative virus concentration was estimated as mentioned above.

### Statistical analysis

All statistical analyses were performed in R (www.r-project.org)^33^. To visualise the effect of *Wolbachia* on viral titre over time, three-parameter asymptotic exponential growth curves were fitted to the viral titre using the R function ‘nls()’. To compare the statistical differences between growth curves, the likelihood ratio test was used. To test whether *Wolbachia* affects viral titre we used a Type II ANOVA with days-post-infection as a categorical fixed effect.

To analyse the mortality of the flies, a Cox's proportional hazard mixed-effect model was analysed in the ‘coxme’ package^34^, including the fixed treatments *Wolbachia* (with or without), virus (LJV, NFV, or control), and vial ID as a random factor. Fecundity was analysed using a Generalized Linear Mixed Model (GLMM) with a zero inflated negative binomial distribution. The GLMMs were fitted using the ‘glmmTMB()’ function in the ‘glmmTMB’ package^35^, including the fixed effects of *Wolbachia* (with or without), virus (LJV, NFV, or control), period post-infection and vial ID as a random factor. The Wald χ^2^ test with a type II sum of squares was used to estimate the significant effects of the models using the ‘Anova()’ function in the ‘car’ package^36^. Interactions that were not significant or of biological interest were removed from the models. Multiple pairwise comparisons with adjusted p-values were performed using the Tukey method in the ‘emmeans’ package. 95% confidence intervals in the plots were estimated by non-parametric bootstrapping (observations were resampled with replacement 1000 times).

## Data Availability

All data generated or analysed during this study are included in this published article and its supplementary information files.
